# Three year outcomes in infants with a family history of autism and/or attention deficit hyperactivity disorder

**DOI:** 10.1002/jcv2.12189

**Published:** 2023-08-02

**Authors:** Tony Charman, Greg Pasco, Alexandra Hendry, Tessel Bazelmans, Nisha Narvekar, Amy Goodwin, Hanna Halkola, Mary Agyapong, Rebecca Holman, Jannath Begum Ali, Mutluhan Ersoy, Mark H. Johnson, Andrew Pickles, Emily J. H. Jones, The STAARS Team

**Affiliations:** ^1^ Institute of Psychiatry, Psychology & Neuroscience King's College London London UK; ^2^ Department of Experimental Psychology University of Oxford Oxford UK; ^3^ Centre for Brain and Cognitive Development Birkbeck University of London London UK; ^4^ Department of Psychology Kastamonu University Kastamonu Turkey; ^5^ Department of Psychology University of Cambridge Cambridge UK

**Keywords:** ADHD, autism, early childhood, infancy, latent profile analysis

## Abstract

**Background:**

Most research on early outcomes in infants with a family history (FH) of autism has focussed on categorically defined autism, although some have language and developmental delays. Less is known about outcomes in infants with a FH of attention deficit hyperactivity disorder (ADHD).

**Methods:**

Infants with and without a FH of autism and/or ADHD, due to a first‐degree relative with either or both conditions, were recruited at 5 or 10 months. Three year outcomes were characterised using latent profile analysis (LPA) across measures of cognitive ability, adaptive functioning and autism, ADHD and anxiety traits (*n* = 131). We additionally ran an LPA using only autism and ADHD measures, and the broader LPA in an independent cohort (*n* = 139) and in both cohorts combined (*n* = 270).

**Results:**

A Low Developmental Level + High Behavioural Concerns class had elevated autism, ADHD and anxiety scores, low cognitive and adaptive function, and included all but one child with autism. A Low Developmental Level + Typical Behaviour class had average cognitive ability and typical behaviour but low adaptive function. A Typical Developmental Level + Some Behavioural Concerns class had average cognitive and adaptive function but slightly elevated behaviour scores. A High Developmental Level + Typical Behaviour class had above average cognitive ability and typical behaviour. All four LPAs identified classes characterised by combinations of either, or both, Low Development Level and elevated behaviour scores, as well as a typically developing class. No classes had elevated autism or ADHD traits in isolation.

**Conclusions:**

Some infants with a FH of autism or ADHD have atypical developmental and behavioural outcomes, but do not show strong autism or ADHD traits in isolation. The field needs to recalibrate aims and methods to embrace the broader transdiagnostic pattern of outcomes seen in these infants.

## Graphical abstract

We used a broad range of outcome measures to identify the ‘natural categories’ of early atypical outcome in infants with family history of autism or ADHD. Some infants with a family history of autism or ADHD have neurodevelopmental profiles characterised by combinations of either, or both, low developmental level and atypical behaviour but not elevated autism or ADHD traits in isolation. The field needs to adopt a transdiagnostic approach in the study of early development in autism and ADHD in order to develop fit‐for‐purpose developmental models and translational opportunities.

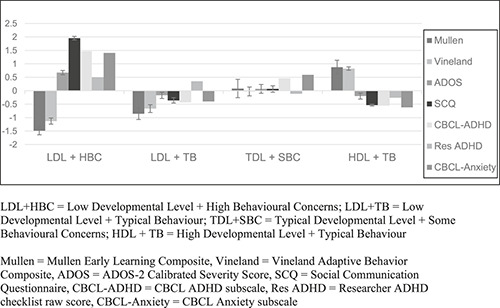

**Key points**

- We used a broad range of outcome measures to identify the ‘natural categories’ of early atypical outcome in infants with FH of autism and/or ADHD.
- Some infants with a FH of autism and/or ADHD have neurodevelopmental profiles characterised by combinations of either, or both, low developmental level and atypical behaviour but not elevated autism or ADHD traits in isolation.
- The field needs to adopt a transdiagnostic approach in the study of early development in autism and ADHD in order to develop fit‐for‐purpose developmental models and translational opportunities.

## INTRODUCTION

Autism is a strongly heritable condition (Tick et al., 2016). Against a population prevalence of ∼1%–2% (Maenner et al., 2020) sibling recurrence rates in clinically ascertained cohorts are approximately 10% (Hansen et al., 2019). Attention deficit hyperactivity disorder (ADHD) is also highly heritable (Thapar & Cooper, 2016). Its prevalence is ∼3%–5% (Polanczyk et al., 2015) but clinically‐ascertained sibling recurrence rates for later‐born siblings are also ∼10% (Miller, Musser, et al., 2019). Autism and ADHD commonly co‐occur at both the clinical and the trait level (Hollingdale et al., 2020; Miller, Musser, et al., 2019). Twin and family studies show moderate shared heritability between the two conditions (Ghirardi et al., 2019; Taylor et al., 2013). Consistent with this, siblings of children with autism have elevated rates of ADHD and, conversely, siblings of children with ADHD have elevated rates of autism (Ghirardi et al., 2019; Jokiranta‐Olkoniemi et al., 2016; Miller, Musser, et al., 2019). Understanding the aetiology of this co‐occurrence has important implications for science and clinical practice (Thapar et al., 2017). Prospective infant designs may provide new insights into this co‐occurrence. Infants are not recruited based on an existing diagnosis of either, or both, autism and ADHD but on the basis of family history (FH). Studying development in infancy before clear manifestations of the behaviours that define autism and ADHD have emerged may help us to identify shared and distinct neurodevelopmental pathways to each condition, both in isolation and in combination (Johnson et al., 2015).

In prospective studies of infants with a FH of autism, recurrence rates of autism are ∼20% (Messinger et al., 2015; Ozonoff et al., 2011). However, there is also an increased rate of broader atypical outcomes beyond an autism diagnosis. Subclinical autism traits, and lower cognitive, language and adaptive ability characterise ∼20% of infants who do not have an autism diagnosis (Charman et al., 2017; Marrus et al., 2018; Messinger et al., 2013; Ozonoff et al., 2014). Few studies have reported the rates of intellectual disability and language delay in prospectively‐studied infants who have autism, but group‐level data from large cohorts suggests these rates are significant (e.g., Table 2, Zwaigenbaum et al., 2021; Figure 2, Messinger et al., 2015).

Fewer studies have reported on early developmental outcomes in prospectively‐studied infants with a FH of ADHD, and findings are more preliminary (Johnson et al., 2015). One recent study of infants with a FH of autism or ADHD characterised clinical outcomes at 3 years. Miller et al. (2020) used latent profile analysis (LPA) of observation and parent‐report measures of autism and ADHD traits and identified three outcome classes: an autism class (with both high autism and ADHD traits), an ADHD class (with high ADHD traits but low autism traits), and a typically developing class. In the same cohort, Reetzke et al. (2021) reported clinically derived outcome groups, comprising those with an autism diagnosis and those with ‘ADHD concerns’ based on a combination of researcher clinical best estimate (CBE) and parent and teacher report of ADHD traits. Miller et al. (2020; Table 3) show high correspondence between the LPA data‐derived autism class and a CBE autism judgement but only moderate correspondence between the ADHD classifications, with significant proportions of those identified in the LPA ADHD class having a CBE of autism or no concerns. To date, no study has reported on broader developmental and behavioural outcomes in a cohort of infants with a FH of ADHD.

### The current study

The extant literature on infants with an autism FH, and the wider literature on family recurrence of neurodevelopmental conditions, suggests that family liability is not specific to autism or to ADHD. Transdiagnostic approaches to understanding early developmental psychopathology are important due to the shared liability factors and high co‐occurrence of these conditions but also because they reflect presentations seen in clinical settings (Astle et al., 2022; Talbott & Miller, 2020; Thapar et al., 2017). In line with this, in the current study we characterised outcome in infants at elevated familial likelihood of autism and ADHD, due to having a first‐degree relative with either or both conditions, by classes defined using LPA across measures of cognitive ability, adaptive behaviour and early autism, ADHD and anxiety traits. We also conducted an analysis similar to Miller et al. (2020) using only autism and ADHD measures and replicated our broader LPA in an independent cohort and in both our cohorts combined. We expected to identify a class of children with autism and broader atypical development and behaviour. We examined whether additional group(s) with atypical outcomes existed and if these showed predominantly elevated early ADHD traits or broader developmental and behavioural difficulties.

## METHODS

### Participants

Infants at either 5 or 10 months of age were enroled if they had a first‐degree relative with a community clinical diagnosis of autism, a first‐degree relative with a community clinical diagnosis of ADHD or elevated ADHD traits, or both. Parental report of an existing clinical diagnosis of autism and/or ADHD in an older sibling (proband) was the most common route. Some parents reported that they themselves had a diagnosis of either condition or they or their older child had suspected ADHD following which screening with a short version of one the Conners suite of measures was employed (see Supplementary Materials Appendix S1, Table S1 and Table S2). Comparison infants with no FH of either condition and a typically developing older sibling were also recruited. Inclusion criteria also included full‐term birth (gestational age >36 weeks) and no known medical or developmental condition. Informed written consent was provided by parents/carers. The total sample (*n* = 161) comprised 80 infants with a first‐degree relative (family history (FH)) with autism only (FH‐Autism), 31 infants with a first‐degree relative with ADHD only (FH‐ADHD), 21 infants with first‐degree relatives with both autism and ADHD (FH‐Autism + ADHD), and 29 infants with no first degree relative with either condition (typical likelihood (TL)) (Table S3). Of these, 131 infants (81.4%) had at least one outcome measure used in the LPA (see below) and are included in the current analysis: 67 FH‐Autism, 26 FH‐ADHD, 16 FH‐Autism + ADHD, and 22 TL (Table 1).

**TABLE 1:** Participant demographic characteristics by family history (FH) sampling frame.

	FH‐Autism	FH‐Autism + ADHD	FH‐ADHD	TL
	*N* = 67	*N* = 16	*N* = 26	*N* = 22
	*N* (%)	*N* (%)	*N* (%)	*N* (%)
Sex				
Male	32 (48%)	11 (69%)	15 (58%)	13 (59%)
Female	35 (52%)	5 (31%)	11 (42%)	9 (41%)
*Age in months*				
Mean (SD)	37.21 (1.46)	37.19 (1.52)	37.35 (2.69)	36.79 (1.78)
Ethnicity (maternal)				
White/European/Irish	53 (83%)	14 (93%)	24 (92%)	17 (85%)
Asian/African/African‐caribbean/Mixed heritage	11 (17%)	1 (7%)	2 (8%)	3 (15%)
Maternal education				
Up to high school/Further education	19 (30%)	8 (53%)	8 (31%)	1 (5%)
University degree or higher	45 (70%)	7 (47%)	18 (69%)	19 (95%)

Abbreviations: FH‐Autism, autism family history; FH‐Autism + ADHD, autism + ADHD family history; FH‐ADHD, ADHD family history; TL, Typical likelihood.

### Three year outcome measures

The Mullen Early Learning Composite (ELC) (Mullen, 1995) – derived from Expressive language, Receptive language, Visual reception and Fine motor subscales – and the Vineland‐II Adaptive Behaviour Composite (ABC) (Sparrow et al., 2005) – derived from Socialization, Communication, Daily Living Skills and Motor domains – were administered as measures of cognitive ability and adaptive functioning, respectively.

The Autism Diagnostic Observation Schedule‐2 (ADOS‐2; Lord et al., 2012), Autism Diagnostic Interview‐Revised (ADI‐R; Lord et al., 1994) and the Social Communication Questionnaire (SCQ; Rutter et al., 2003) were administered. Autism traits were also measured with the Social Responsiveness Scale 2‐Preschool Form (SRS‐2; Constantino & Gruber, 2012). Best estimate Diagnostic and Statistical Manual of Mental Disorders, 5th Edition (DSM‐5) clinical diagnosis of Autism Spectrum Disorder was informed by, but not dependent on, scores on the ADOS‐2, ADI‐R, SCQ, Vineland‐II, and Mullen, and researcher observations and parent‐reported information, by experienced researchers (TC, GP).

Emerging ADHD and anxiety traits were measured using the parent‐report DSM subscales of the Child Behaviour Checklist‐Preschool (CBCL‐P 1.5–5; Achenbach & Ruffle, Achenbach and Rescorla (2000)). An observational measure of early ADHD behaviours (attentiveness, activity level, and inhibition to objects/the environment; scored on a 7 point Likert scale) was completed using consensus coding by researchers based on observations made across the visit.

### Statistical analysis

We conducted LPA using continuous indicator variables to identify homogeneous classes based on the following 3 years outcome variables: Mullen ELC, Vineland ABC, ADOS‐2 CSS, SCQ total score, researcher early ADHD observation total score, and CBCL ADHD and anxiety subscale raw scores. Variables were modelled, conditional on latent class, using Poisson distributions for all variables except SCQ where overdispersion required a negative binomial distribution. Latent profile analysis was performed using the *gsem* command in Stata 16 (StataCorp., 2019) on the whole sample with at least one of the seven outcome measures available (*n* = 131; 122 children had ≥4 measures, 4 ≥ 3, 3 ≥ 2, and 2 children 1 measure only). Models were estimated using maximum likelihood to account for data missing at random. To select the “best fitting” solution we examined conventional likelihood‐based (Bayesian information criterion (BIC)) and classification‐based (Integrated Classification Likelihood (ICL); entropy) fit statistics (Henson et al., 2007); the proportion of participants represented in each class; and the extent to which classes captured clinically meaningful subgroups of participants (Nylund et al., 2007). Individuals were assigned to classes based on the maximum aposterior probability of class membership (MAP).

We named the outcome classes in terms of developmental level (cognitive ability and adaptive function) and behaviour (autism, ADHD and anxiety traits) – namely the indicator variables from which they were derived. We compared the scores of the outcome classes using ANOVA with post‐hoc Tukey‐Kramer corrections to account for unequal cell sizes and tested sex differences in class assignment using chi‐square tests followed by post‐hoc residuals adjusted for the sample size of each group (Kirk, 1998). The high values of MAP we report made more complex multi‐step post‐assignment analysis methods unnecessary.

## RESULTS

Three‐ and four‐class solutions had similar entropy values (0.82 and 0.80, respectively) and the best fit statistics (BIC = 4486.24, ICL = 4537.94 and BIC = 4484.21, ICL = 4555.60, respectively; Table S4). We chose the four‐class solution as providing the most robust and clinically meaningful distribution of classes, with a minimum class size comprising 15.6% (*n* = 20) of the sample and average MAP values for each class all ≥0.83. Table S5 shows the correlations between the class indicator variables and R‐squared values from regressing each indicator onto the set of classes.

**FIGURE 1:**
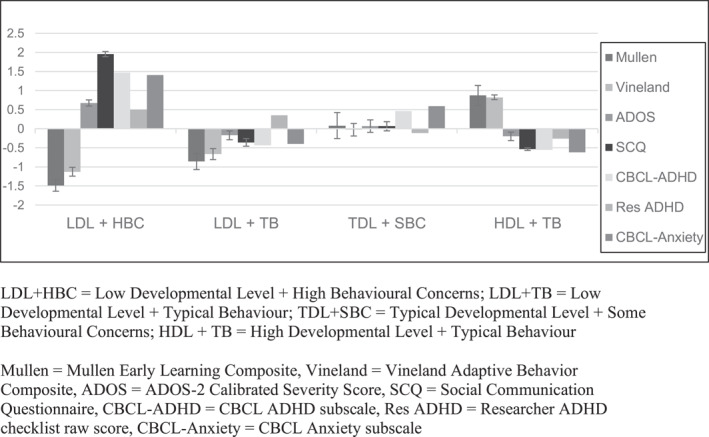
Profile on indicator variables of latent profile analysis (LPA) outcome classes. Y‐Axis scale is z‐score derived separately for each measure from the current sample so all measures are similarly scaled to provide a profile across the measures.

Scores of the four‐classes on the outcome measures used to derive the classes are shown in Table 2 ^1^ and Figure 1. Based on the pattern across the measures we labelled the classes: *Low Developmental Level + High Behavioural Concerns* (LDL + HBC; *n* = 20, 16%), *Low Developmental Level + Typical Behaviour* (LDL + TB; *n* = 23, 19%), *Typical Developmental Level + Some Behavioural Concerns* (TDL + SBC; *n* = 33, 24%), and *High Developmental Level + Typical Behaviour* (HDL + TB; *n* = 55, 42%).

**TABLE 2:** 3 Year developmental and behavioural characteristics by latent profile analysis (LPA) outcome class – main cohort.

	LDL + HBC^1^ class	LDL + TB^2^ class	TDL + SBC^3^ class	HDL + TB^4^ class	ANOVA	
	*N* = 20	*N* = 23	*N* = 33	*N* = 55		
Measure	*M* (SD)	*M* (SD)	*M* (SD)	*M* (SD)		Post‐hocs
Measures used to derive LPA outcome classes						
Mullen ELC^a^	84.38 (11.96)	96.57 (10.72)	114.84 (8.70)	130.19 (8.83)	F(113,3) = 119.17***	4 > 1, 2, 3; 3 > 1, 2; 2 > 1
Vineland ABC^a^	81.52 (10.60)	87.14 (8.27)	94.86 (7.42)	105.17 (7.58)	F(105,3) = 43.47***	4 > 1, 2, 3; 3 > 1, 2
ADOS‐2 CSS^b^	3.11 (2.19)	1.83 (1.19)	2.19 (1.38)	1.78 (1.30)	F(119,3) = 3.97**	1 > 2, 4
SCQ^c^	19.63 (7.50)	3.48 (1.99)	6.43 (3.76)	2.25 (1.70)	F(109,3) = 100.62***	1 > 2, 3, 4; 3 > 2, 4
CBCL ADHD^d^	65.24 (7.50)	51.19 (2.66)	55.48 (5.56)	51.02 (2.85)	F(112,3) = 46.39***	1 > 2, 3, 4; 3 > 2, 4
Researcher ADHD^c^	12.67 (2.69)	12.33 (2.24)	11.27 (1.71)	10.96 (2.20)	F(104,3) = 3.50*	1 > 4
CBCL anxiety^d^	64.41 (10.08)	50.95 (1.83)	57.33 (7.55)	50.27 (1.02)	F(112,3) = 35.24***	1 > 2, 3, 4; 3 > 2, 4
Other measures (not used in the LPA analysis)						
SRS^d^	71.47 (13.79)	45.75 (4.83)	52.28 (8.73)	42.17 (3.57)	F(103,3) = 63.66***	1 > 2, 3, 4; 3 > 2, 4
ADI‐social^c^	12.37 (7.33)	2.57 (3.16)	2.81 (3.20)	0.82 (1.01)	F(118,3) = 47.93***	1 > 2, 3, 4
ADI‐comm^c^	8.74 (5.32)	2.83 (3.65)	2.48 (2.61)	0.51 (1.00)	F(118,3) = 34.64***	1 > 2, 3, 4; 2, 3 > 4
ADI‐RRB^c^	3.68 (2.31)	0.52 (0.73)	1.61 (1.86)	0.24 (0.69)	F(118,3) = 29.85***	1 > 2, 3, 4; 3 > 2, 4

*Note*: 1, 2, 3, 4 – Class labels for ANOVA Tukey‐Kramer corrected post‐hocs.

Abbreviations: ABC, Vineland Adaptive Behaviour Composite; ADI, Autism Diagnostic Interview; ADOS‐2 CSS, ADOS‐2 Calibrated Severity Score; CBCL, Child Behaviour Checklist; Comm, Communication; ELC, Mullen Early Learning Composite; HDL + TB, High Developmental Level + Typical Behaviour; LDL + HBC, Low Developmental Level + High Behavioural Concerns; LDL + TB, Low Developmental Level + Typical Behaviour; RRB, Repetitive and Restricted Behaviours; SCQ, Social Communication Questionnaire; SRS, Social Responsiveness Scale; TDL + SBC, Typical Developmental Level + Some Behavioural Concerns.

^a^standard score.

^b^ADOS‐2 Calibrated Severity Score.

^c^raw score.

^d^T‐score.

*p < 0.05, **p < 0.01, ***p < 0.001.

ANOVAs and Tukey‐Kramer post‐hocs for class differences are in line with class identification. The LDL + HBC class had elevated autism, ADHD and anxiety scores, low cognitive ability and adaptive function. The LDL + TB class had low autism, ADHD and anxiety scores, average cognitive ability but below average adaptive function. The TDL + SBC class had average cognitive ability and adaptive function but slightly elevated scores on the SCQ and the CBCL ADHD and anxiety subscales. The HDL + TB class had low autism, ADHD and anxiety scores, above average cognitive ability and average adaptive function. When considered in terms of the proportion of each class falling above or below clinical thresholds (>1SD for standardised scores and T‐scores; above the autism threshold on the ADOS‐2 and SCQ^2^) the pattern was similar (Table 3). Thirteen children met diagnostic criteria for autism – 12 were identified in the LDL + HBC class and 1 in the TDL + SBC class.^3^

**TABLE 3:** Number and percentage of each latent profile analysis (LPA) class in atypical range on 36 months characterisation measures – main cohort.

	LDL + HBC class	LDL + TB class	TDL + SBC class	HDL + TB class
Measure	N = 20	N = 23	N = 33	N = 55
Mullen ELC <85	7 (44%)	3 (13%)	0 (0%)	0 (0%)
Vineland ABC <85	12 (71%)	9 (41%)	0 (0%)	1 (2%)
ADOS CSS ≥4	7 (39%)	3 (13%)	7 (23%)	9 (18%)
SCQ score ≥12	15 (88%)	0 (0%)	2 (8%)	0 (0%)
ADHD rating	N/A	N/A	N/A	N/A
CBCL ADHD T‐score ≥60	15 (88%)	1 (5%)	5 (20%)	2 (4%)
CBCL anxiety T‐score ≥60	12 (71%)	0 (0%)	10 (38%)	0 (0%)

Abbreviations: ABC, Vineland Adaptive Behaviour Composite; ADOS‐2 CSS, ADOS‐2 Calibrated Severity Score; CBCL, Child Behaviour Checklist; ELC, Mullen Early Learning Composite; HDL + TB, High Developmental Level + Typical Behaviour; LDL + HBC, Low Developmental Level + High Behavioural Concerns; LDL + TB, Low Developmental Level + Typical Behaviour; N/A, Not applicable; SCQ, Social Communication Questionnaire; TDL + SBC, Typical Developmental Level + Some Behavioural Concerns.

Table 4 shows the association between the derived classes and the autism and ADHD FH sampling frame and sex. All children in the LDL + HBC and LDL + TB classes were from the FH groups. Children from the TDL + SBC and HDL + TB classes were from all the sampling groups. Boys were over‐represented in the LDL + HBC and LDL + TB classes but these differences were non‐significant (overall chi‐square *p* = 0.18).

**TABLE 4:** Latent profile analysis (LPA) outcome class by familial likelihood group and sex – main cohort.

		LDL + HBC class	LDL + TB class	TDL + SBC class	HDL + TB class	
		*N* = 20	*N* = 23	*N* = 33	*N* = 55	
Familial likelihood group						
TL	N (%)	0 (0%)	0 (0%)	4 (18%)	18 (82%)	22
FH‐Autism	N (%)	14 (21%)	15 (22%)	16 (24%)	22 (33%)	67
FH‐Autism + ADHD	N (%)	5 (31%)	2 (13%)	6 (38%)	3 (19%)	16
FH‐ADHD	N (%)	1 (4%)	6 (23%)	7 (27%)	12 (46%)	26
						131
*Sex*		N (%)	N (%)	N (%)	N (%)	Total N
Male		13 (65%)	16 (70%)	17 (52%)	25 (45%)	71
Female		7 (35%)	7 (30%)	16 (48%)	30 (55%)	60
						131

Abbreviations: LDL + HBC, Low Developmental Level + High Behavioural Concerns; LDL + TB, Low Developmental Level + Typical Behaviour; TDL + SBC, Typical Developmental Level + Some Behavioural Concerns; HDL + TB, High Developmental Level + Typical Behaviour.

We ran a second LPA using only measures of autism and ADHD (ADOS‐2 CSS, SCQ total score, researcher ADHD observation total score, and CBCL ADHD raw score). The three‐class solution provided the most robust and clinically meaningful distribution of classes, with a minimum class size comprising 17.0% (*n* = 20) of the sample and average MAP values for each class all ≥0.84 (Table S6). The identified classes were: *High Behavioural Concerns* (HBC; *n* = 20, 17%), *Slight Behavioural Concerns* (SBC; *n* = 72, 54%), and *Typical Behaviour* (TB; *n* = 37, 29%). These 3 classes are similar to those identified by the LPA with a broader set of indicator variables except that the class with LDL + TB was not identified, reflecting the fact that Mullen and Vineland were not indicators. The HBC class had elevated autism and ADHD scores on the measures used to derive the classes and included all 12 children^4^ with an autism diagnosis. However, when examining scores on the other measures not used to derive the classes they also had elevated anxiety scores, low cognitive ability and low adaptive function (Table S7).

We repeated the broader LPA analysis in an independent cohort of *n* = 139 infants with a FH of autism and/or ADHD with outcome data at 3 years using the Mullen ELC, Vineland ABC, ADOS‐2 CSS, SCQ total score, and CBCL ADHD and anxiety subscale raw scores.^5^ The four‐class solution provided the most robust and clinically meaningful distribution of classes, with a minimum class size comprising 17.0% (*n* = 20) of the sample and average MAP values for each class all ≥0.91 (Table S8). Based on the pattern across the measures we labelled the classes: *Low Developmental Level + High Behavioural Concerns* (LDL + HBC; *n* = 21, 15%), *Typical Developmental Level + High Behavioural Concerns* (TDL + HBC; *n* = 27, 19%), *Low Developmental Level + Typical Behaviour* (LDL + TB; *n* = 20, 14%), and *High Developmental Level + Typical Behaviour* (HDL + TB; *n* = 71, 51%) (See Appendix S2, Table S9, Table S10, Table S12 and Figure S1). Sixteen children met diagnostic criteria for autism: 10 of these were identified in the LDL + HBC class, 5 in the TDL + HBC class, and 1 in the LDL + TB class. Girls were over‐represented in the HDL + TB class (*p* < 0.01) and boys in the LDL + HBC (*p* < 0.01) and LDL + TB (*p* < 0.05) classes (Table S11).

We then repeated the broader LPA in the combined sample of both cohorts (*n* = 270). Similar LDL + HBC and HDL + TB classes to those described above emerged. A second atypical *Low Developmental Level + Typical Behaviour* (LDL + TB) class had slightly low cognitive ability and adaptive function but did not have elevated scores on autism, ADHD or anxiety measures. A *Typical Developmental Level + Some Behavioural Concerns* (TDL + SBC) class had slightly elevated scores on ADHD and anxiety measures and on the SCQ but not the ADOS, high average cognitive ability and average adaptive function. These patterns were reflected both in terms of group means (Table S13, Figure S2) and the proportion of the class scoring above or below the 1SD and clinical thresholds (Table S14; Appendix S3, correlations shown in Table S16). Twenty eight children met diagnostic criteria for autism: 19 in the LDL + HBC class, 8 in the TDL + SBC class, and 1 in the LDL + TB class. Girls were over‐represented in the HDL + TB class (*p* < 0.01) and boys in the LDL + HBC (*p* < 0.05) and LDL + TB (*p* < 0.05) classes (Table S15).

## DISCUSSION

We used a data‐driven approach to identify outcome classes across measures of cognitive ability and adaptive functioning, and autism, ADHD and anxiety traits, at 3 years in infants with a FH of autism or ADHD, or both. We identified a class with elevated autism, ADHD and anxiety scores, low cognitive ability and adaptive function, which included most children with an autism diagnosis. A second atypical class had low autism, ADHD and anxiety scores, average cognitive ability but below average adaptive functioning; and a third atypical class had average cognitive ability and adaptive function but slightly elevated behavioural scores. A typically developing class with above average cognitive ability was also identified.

In an independent cohort, we also identified three atypical classes with overlapping but slightly different profiles: two with high behaviour scores, one with low cognitive ability and one average cognitive ability, and a third class with low cognitive ability but typical behaviour, although this class had elevated ADOS scores most likely reflecting their developmental delay. Boys were over‐represented in the atypical outcome classes compared to girls and whilst this did not reach significance in our primary analysis it did in the independent cohort and both cohorts combined. When an LPA was conducted in the combined sample, one typically developing class with above average cognitive ability was found. Atypical classes comprised one with LDL + HBC that included most children with autism; another with slightly low cognitive ability and adaptive behaviour; and a third atypical classes with typical cognitive ability and slightly elevated ADHD, anxiety and autism scores. Across all analyses no outcome class with elevated autism or ADHD traits in isolation was found.

When only measures of autism and ADHD were used as class indicators we identified a similar (largely overlapping) class that had elevated autism and ADHD behaviours but also elevated anxiety and below average cognitive ability and adaptive function on measures that were not used to derive the LPA classes. Even when considering this class only in terms of the autism and ADHD indicator variables used in the LPA they had both elevated autism and ADHD scores. One other class had very slightly elevated scores on the parent‐report SCQ and CBCL‐ADHD subscale and another class typical behaviour. This pattern differs from Miller et al. (2020) who used a similar data‐driven LPA approach to characterise outcome classes at 3 years in infants with a FH of autism or ADHD. Miller et al. identified an autism class, which also had elevated ADHD scores and low cognitive ability (p.1327 and Table S2, Miller et al., 2020), an ADHD class who had slightly but not clinically elevated autism scores on the SCQ and the ADOS‐2 and average cognitive ability, and a typically developing class.^6^ We did not identify a class with elevated ADHD scores only, even in the LPA model using only autism and ADHD measures to derive classes. There are several differences between the studies that may in part explain these discrepancies. In terms of recruitment, Miller et al. (2020) created separate ADHD and autism FH groups and infants with a FH of both autism and ADHD were allocated to the autism FH group, whereas we treated FH of autism and ADHD alone and in combination independently. Whilst both studies used the ADOS and the SCQ as autism indicator variables in their LPA, Miller et al. used different researcher‐ and parent‐rated measures of ADHD to the current study.

### Approaches to characterising outcomes in infants with a family history of autism and/or attention deficit hyperactivity disorder

Although we found moderately high recurrence rates of autism in our samples (10% in the primary sample, 12% in the independent sample), autism in isolation is not the primary atypical outcome in early childhood of infants with an autism FH (with or without an ADHD FH). This is neither a new nor a surprising finding. In clinical samples both autism and ADHD commonly co‐occur with other neurodevelopmental and neuropsychiatric conditions. Studies of older siblings and family members show not only that autism and ADHD commonly co‐occur (Miller, Musser, et al., 2019; Musser et al., 2014) but also that in siblings of children with autism and ADHD other neurodevelopmental and neuropsychiatric conditions including anxiety, conduct disorder, intellectual disability and language delay are also common (Jokiranta‐Olkoniemi et al., 2016, 2019). This is consistent with the atypical outcome classes we identified in all four analyses conducted where a combination of either, or both, broader atypical behaviour and low cognitive and/or adaptive function was seen across the classes. In studies of autism FH infants many of those who have autism at 3 years have low cognitive ability and adaptive behaviour (Messinger et al., 2015; Zwaigenbaum et al., 2021). Consistent with previous studies, both in infants with autism and those without (Messinger et al., 2013, 2015), we found boys to be over‐represented in the classes with lower developmental level and higher behavioural concerns.

In infants with an autism FH who do not have autism ∼20% are characterised by elevated but sub‐clinical autism traits, low cognitive, language and adaptive ability (in combination sometimes referred to as the ‘broader autism phenotype’ or ‘other developmental concerns’), and also elevated rates of emotional and behavioural problems (Charman et al., 2017; Marrus et al., 2018; Messinger et al., 2013; Miller, Iosif, et al., 2019; Ozonoff et al., 2014). In most previous autism FH infant studies, including ours, the group of children who meet diagnostic criteria for autism at 3 years are labelled the ‘autism outcome’ group. Clearly a group of children who do meet the diagnostic criteria for autism exist, and without exception children with an autism diagnosis were in the atypical LPA classes identified here. However, the use of the singular ‘autism’ term to describe this group may inadvertently overshadow their broader profile of developmental and behavioural atypicality, as well as the fact that they share many characteristics with children who may fall just below the clinical threshold for a diagnosis.

We chose to name the outcome classes identified in terms of developmental level (cognitive ability and adaptive function) and behaviour (autism, ADHD and anxiety traits) – namely the indicator variables from which they were derived. Since our focus was on broader transdiagnostic characterisation of developmental and behavioural outcomes in the cohorts at 3 years and not categorical diagnosis we have described the developmental outcomes as ‘low’, ‘typical’ and ‘high’ developmental level and the behavioural outcomes as ‘typical behaviour’ and ‘some’ or ‘high’ ‘behavioural concerns’. These do not equate to clinical classifications and we did not apply a strict threshold for their application – in part because across the LPAs we ran classes often had slightly different profiles of scores across the individual measures. We have used terminology and concepts from the developmental psychopathology/child psychiatry field to describe the outcome classes since ‘developmental level’ and ‘behavioural concerns’ map directly onto the indicator measures used to form LPA classes (Mullen and Vineland, and ADOS, SCQ and CBCL, respectively) and align with both previous literature and conventional clinical usage. However, we acknowledge the changing conceptual and linguistic landscape in the field and the outcome groups could alternatively be labelled as showing moderate or high ‘neurodivergent’ development (Dwyer, 2022; Sonuga‐Barke & Thapar, 2021).

However, inspection of both the mean scores and the proportions of each identified class falling above or below the clinical cut‐points on screening and diagnostic measures, or −/+ 1SD on standardised measures, indicate that the atypical classes identified have partially distinct but also partially overlapping profiles. This approach is similar to that adopted in previous studies (e.g. Charman et al., 2017; Messinger et al., 2013; Miller et al., 2020). Children with elevated autism, ADHD and anxiety traits at this age may not meet the threshold for a clinical condition nor necessarily require intervention or support. Notwithstanding this, across the current analyses between 50% and 60% of the infants with a FH of autism and/or ADHD were identified in one of the atypical classes. Given the FH nature of the cohorts and the fact that here we characterise outcomes only up to age 3 years it will be important to study and monitor these children as they grow older.

### Implications for the study of family history infants

The findings of the present analysis go beyond nomenclature and should influence the motivation, design, analysis and interpretation of future FH studies. For example, the broader transdiagnostic pattern of atypical neurodevelopmental outcomes we have identified has important implications for studies that aim to identify early biomarkers and endophenotypes of autism (Johnson et al., 2015; Jones et al., 2014; Szatmari et al., 2016; Wolff & Piven, 2021). Although many infant signs and biomarkers have been mooted (and some patented) as predicting later autism, if the ‘natural categories’ of early outcome in infants with a FH are broader – both across autism versus other behavioural phenotypes (here, ADHD and anxiety) and across behavioural versus developmental outcomes (here, cognitive ability and adaptive behaviour) – then there needs to be a re‐calibration of aims and methods. These cautions also apply to mechanistic interpretations where infant experimental signals are taken to be precursors of later social communication, repetitive or sensory behaviours, without addressing the extent to which they may be confounded by cognitive ability, adaptive function or later emerging ADHD or anxiety traits. This may motivate research groups, including ours, to re‐analyse previously published findings to test the specificity of prediction to autism versus other atypical neurodevelopmental outcomes.

Other important future goals include examining whether there is greater differentiation of the developmental and behavioural phenotypic pattern we have observed at age 3 years as children are followed to mid‐childhood (e.g. Shephard et al., 2017) and exploring the age and the indicators in the infancy period that might identify children who will later develop this presentation (e.g. Kostyrka‐Allchorne et al., 2020).

### Strengths and limitations

The study has several strengths. In a moderate size sample of infants with an autism and/or ADHD FH followed to age 3 years we have used a data‐driven approach to derive outcomes employing a broad range of developmental and behavioural measures rather than only autism symptoms. We replicated our approach in an independent sample and in both samples combined. However, this latter sample was recruited based on an established FH of autism and while information on FH of ADHD was collected this was not an integral part of the recruitment. Limitations include that we only studied outcomes through to 3 years. The characterisation of ADHD, anxiety and other neurodevelopmental outcomes – as well as autism itself (Ozonoff et al., 2018) – is incomplete at this age (Rocco et al., 2021) and further work is needed to develop and validate accurate and reliable measures of different forms of emerging developmental psychopathology in the early years. We have previously followed autism FH infants to mid‐childhood and found elevated levels of ADHD and anxiety traits both in those children with an autism diagnosis and those without (Shephard et al., 2017). We did not obtain sufficient information at 3 years to ascertain ADHD diagnoses. A number of measures employed in the current study are parent‐report and suffer from potential shared methods and reporting bias limitations. Examination of the regressions of LPA indicator variables onto the LPA class solution shows that these measures (and also the direct Mullen assessment) were most strongly correlated with the identified classes, and that whilst the observational measures (ADOS and researcher ADHD ratings) contributed significantly the correlations were notably weaker. Another, related limitation of reliance on parental report questionnaires is the ability both of parents and the questionnaires themselves to distinguish between specific behaviours that may be manifestations of different emerging difficulties (see Hus et al., 2013). Finally, our researcher observational measure of early ADHD behaviours was not a standard measure, observers were not blind to FH, and whilst we conducted researcher consensus ratings no inter‐rater reliability data was collected.

### Conclusions

Family history, twin and genetic studies suggest that family liability is not specific to autism or to ADHD. There is overlap in early genetic liabilities related to the later expression of both phenotypes (Constantino et al., 2021), and autism and ADHD (and other early neurodevelopmental phenotypes) are underpinned by partially overlapping and partially separate developmental processes (Johnson et al., 2015, 2021; Miller et al., 2020). Much progress has been made in understanding the early development of autism, including from infant FH studies, and recent progress is apparent in the early ADHD field (Miller et al., 2023). However, if as we demonstrate here, the ‘natural categories’ of atypical outcome of infants with a FH of autism and/or ADHD are not the autism and ADHD phenotypes in isolation, but broader atypical developmental and behavioural outcomes, then the field needs to embrace this challenge. This concept is similar to that identified previously by Gillberg (2010) as ‘ESSENCE’ (Early Symptomatic Syndromes Eliciting Neurodevelopmental Clinical Examinations). Gillberg argued that early manifestations of neurodevelopmental conditions (seen in clinic) are commonly non‐specific, affecting motor, cognitive, communicative and social development, as well as sleep, feeding and behavioural regulation.

Research on developmental models and approaches to clinical translation should adopt a transdiagnostic approach in developmental studies of early autism and ADHD. Initial suggestions include the need to explicitly test the ‘phenotype‐specificity’ of early infant neurodevelopmental markers of later outcomes; and the development of pre‐emptive interventions that promote broader developmental competencies and outcomes, rather than targeting emergent symptoms of specific neurodevelopmental conditions (Constantino et al., 2021; Manzini et al., 2021). One critical question is whether at this age atypical development is truly transdiagnostic, akin to the ESSENCE concept, and may only differentiate later in development; or whether developmental ability, autism, ADHD and anxiety are separable but simply cluster or co‐occur (in this cohort, with the current measures) at this developmental timepoint. Experimental measures that assay underlying neuroendophenotypic processes, continued study of these cohorts as they age, and item‐level network analysis approaches to measurement, may all in future studies help answer these questions.

## AUTHOR CONTRIBUTIONS

TC, MHJ, AP and EJHJ obtained funding and designed the study. AH, TB, NN, AG, HH, MA, RH, and ME were involved in conducting the study and/or data collection or analysis. TC and AP conducted the data analysis The article was drafted by TC and all authors read, made revisions and approved the final version.

## CONFLICT OF INTEREST STATEMENT

TC has served as a paid consultant to F. Hoffmann‐La Roche Ltd. and Servier; and has received royalties from Sage Publications and Guilford Publications. AP receives royalties from Western Psychological Services, Imperial College Press, and OUP. MJ receives royalties from Wiley‐Blackwell, OUP and MIT Press. EJ is a Joint Editor on JCPP Advances. The remaining authors have declared that they have no competing or potential conflicts of interest.

## ETHICAL CONSIDERATIONS

Ethical approval was granted by the National Research Ethics Service (13/LO/0751 and 08/H0718/76) and the Research Ethics Committee, Department of Psychological Sciences, Birkbeck, University of London.

## Supporting information

Supplementary Material

Figure S1

Figure S2

## Data Availability

Data available following a review of requests as indicated here: https://www.basisnetwork.org/collaboration‐and‐project‐affiliation/.

## ENDNOTES

^1^
Table 2 shows T‐scores for the CBCL measures for descriptive purposes but raw scores were entered into the LPA analysis.

^2^
We used the ≥12 threshold on the SCQ for 3 year olds (Corsello et al., 2007).

^3^
We note that only 48% of children with elevated ADHD scores and 36% of children with elevated anxiety scores had an autism diagnosis.

^4^
One fewer child with autism had at least one of more restrictive list of outcome class indicators.

^5^
The researcher ADHD observation measure was not completed in this cohort.

^6^
We are very grateful to Dr. Meghan Miller for generously sharing unpublished data from Miller et al. (2020).
