# Supplementary material for: Three year outcomes in infants with a family history of autism and/or attention deficit hyperactivity disorder
Source: JCPP Adv. 2023 Aug 2;3(4):e12189. doi: 10.1002/jcv2.12189 (PMC10694531; doi:10.1002/jcv2.12189)
Supplement: Supplementary file 1 — Supplementary Material [file JCV2-3-e12189-s002.docx]

08/05/2023

Three Year Outcomes in Infants with a Family History of Autism and / or Attention Deficit Hyperactivity Disorder (ADHD)

**Supporting Information**

# Appendix S1

# Methods

## Cohort ascertainment – Main cohort (see also Begum-Ali et al, 2022, 2023)

Participants were recruited for a longitudinal study running from 2013 to 2019 from a volunteer database, community flyers, internet adverts and clinical networks. Information about diagnostic status was ascertained through a number of methods. Before families enrolled in the study, a telephone screening form was used to determine the presence of ASD and ADHD in family members. During their infant’s visit to the lab, the parent/caregiver also completed a medical and psychiatric history interview. We asked for medical updates at each study visit and re-administered the medical and psychiatric history interview at the 24 month timepoint. We also requested diagnostic letters and asked parents to complete the DAWBA (Goodman, Ford, Richards, Gatward & Meltzer, 2000), the Conners (Conners, 2008) and the Social Communication Questionnaire (Rutter, Bailey & Lord, 2003) on the family member with a diagnosis and where possible all other family members. This information is used to characterise our sample rather than for exclusionary purposes since, in the UK, NHS clinical diagnoses follow a gold-standard procedure including collation of information from parents, teachers and from in-person assessment that is beyond the scope of this study and more accurate than simple questionnaire measures.

Up to 30% of children with ASD meet criteria for ADHD when directly assessed (Simonoff, Pickles, Charman, Chandler, Loucas & Baird, 2008). In clinical practice, the prevalence of dual diagnosis is in practice much lower (Russell, Rodgers, Ukoumunne & Ford, 2014). Given the nature of the co-occurrence between ASD and ADHD and our longitudinal study, sometimes family members would have a suspected diagnosis of ADHD at study entry that would be confirmed later in the study; on other occasions, a family would enrol on the basis of an ASD diagnosis in an older sibling but by the end of the study, they would report that the same sibling was now undergoing assessment for suspected additional ADHD. Where possible, families who reported suspected ADHD at study entry were screened using a shortened version of the Conners. For siblings (aged less than 6 years), a shortened version of the Conners Early Childhood (Conners, 2008, Conners & Goldstein, 2009) form is used. For siblings (6 years or older), a shortened version of the Conners 3 was used. Thresholds for inclusion were the presence of 6 ADHD traits on either the hyperactivity/impulsivity or inattention scale, and a positive score on the impairment scale. For parents a shortened version of the Conners Adults ADHD Rating Scale (CAARS; Conners et al., 1999) was used. Thresholds for inclusion were the presence of 5 ADHD traits on either the hyperactivity/impulsivity or inattention scale as per DSM-5 guidelines (see Table S1 for categorisation of the cohort). In terms of the use impairment scores, we used a reduced version of the Conners EC and Conners 3 for individuals under 18 and the CAARS for individuals aged 18+ years. The Conners EC and Conners 3 included questions regarding impairment, as such we also included these questions in our screening forms. In comparison, the CAARS (adult questionnaire) did not include questions regarding impairment. In order to maintain consistency of measure, we did not adapt the CAARS to add impairment questions. Of note, at initial contact with participants, parents were asked if there were any diagnoses of ADHD in the immediate family or if they had any concerns about ADHD. It is only if parents reported concerns that the screening process took place. Families who screened positive on this instrument were then included as a confirmed case.

Table S1 *Categorisation of Family History Sampling Framework – Main Cohort*

|  | FH-ASD  *N* = 80 | FH-ADHD  *N* = 31 | FH-ASD+ADHD  *N* = 21 |
| --- | --- | --- | --- |
| Parent reported diagnosis in older sibling | 74 | 8 | 16 |
| Parent reported diagnosis in parent | 3 | 18 | 3 |
| Parent reported diagnosis in both older sibling and parent | 3 | 1 | 1 |
| Screened parent (for ADHD traits) |  | 1 | 1^a^ |
| Screened older sibling (for ADHD traits) |  | 3 |  |

FH-Autism = autism family history, FH-Autism + ADHD = autism + ADHD family history, FH-ADHD = ADHD family history

a Sibling diagnosed with ASD, also screened for ADHD traits

## Cohort ascertainment – Independent cohort

Participants were recruited for a longitudinal study running from 2008 to 2013 from a volunteer database, community flyers, internet adverts and clinical networks. For this cohort recruitment of EL for autism was based on an existing local clinical diagnosis of autism in an older sibling (proband). For all 116 children with an older sibling with a community clinical diagnosis of ASD (hereafter probands) parents completed the Development and Well-Being Assessment (DAWBA: Goodman, Ford, Richards, Gatward, & Meltzer, 2000) and/or the Social Communication Questionnaire (SCQ: Rutter, Bailey, & Lord, 2003). Eighty-nine probands met criteria on both the DAWBA and SCQ. Seven children scored below threshold on the SCQ and one was missing the SCQ, but no exclusions were made due to meeting threshold on the DAWBA and expert opinion. For 19 probands, confirmation of local clinical diagnosis was only available via the SCQ. Screening for possible ASD in the older siblings of the 27 typical likelihood (TL) infants was undertaken using the SCQ, with no child scoring above the instrument cut-off for ASD (>15). For one TL child the SCQ was missing. Medical history review confirmed a lack of ASD within first-degree relatives.

Subsequently to align the family history status with the main cohort we reviewed medical history (updated at each visit) for existing ADHD diagnosis in older siblings and parents. In addition, we also screen older siblings and parents using the Conners Early Childhood and Conners Adults ADHD Rating Scale (CAARS), respectively.

Of the total sample of n = 143 infants, 139 (97.2%) had at least one outcome measure used in the LPA and are included in the current analysis: 82 FH-Autism, 1 FH-ADHD, 31 FH-Autism + ADHD, and 25 TL.

Table S2 *Categorisation of Family History Sampling Framework – Independent Cohort*

|  | FH-ASD  *N* = 82 | FH-ADHD  *N* = 1 | FH-ASD+ADHD  *N* = 31 |
| --- | --- | --- | --- |
| Parent reported diagnosis in older sibling | 81 | 0 | 24 |
| Parent reported ADHD diagnosis in parent | 0 | 1 | 0 |
| Parent reported diagnosis in both older sibling and parent | 1 | 0 | 0 |
| Sibling parent diagnosed with autism and/or ADHD |  | 0 | 5 |
| Sibling diagnosed with autism; parent screened for ADHD traits |  | 0 | 1 |
| Sibling diagnosed with autism and ADHD; parent screened for ADHD traits |  | 0 | 1 |

FH-Autism = autism family history, FH-Autism + ADHD = autism + ADHD family history, FH-ADHD = ADHD family history

Table S3 *Participant Demographic Characteristics by Family History Sampling Frame*

*– Independent Cohort*

**FH-Autism FH-Autism FH-ADHD TL**

**+ ADHD**

*N = 82 N = 31 N = 1 N = 25*

*N (%) N (%) N (%) N (%)*

*Sex*

Male 46 (56%) 16 (52%) 0 (0%) 14 (56%)

Female 36 (44%) 15 (48%) 1 (100%) 11 (44%)

*Age in months*

Mean (SD) 38.65 (1.65) 39.19 (1.74) 38 (0) 38.75 (1.65)

*Ethnicity (maternal)*

White/European/ 65 (79%) 27 (87%) 1 (100%) 21 (84%)

Irish

Asian/African/ 17 (21%) 4 (13%) 0 (0%) 4 (16%)

African-Caribbean/

Mixed Heritage

*Maternal Education*

Up to High School/ 32 (41%) 11 (39%) 0 (0%) 3 (12%)

Further Education

University Degree 47 (59%) 17 (61%) 1 (100%) 22 (88%)

or Higher

FH-Autism = autism family history, FH-Autism + ADHD = autism + ADHD family history, FH-ADHD = ADHD family history, TL = Typical likelihood

Table S4 *Summary of the Broader Latent Profile Analysis (LPA) Models – Main Cohort*

Number of Entropy BIC^a^ ICL^b^ Relative prevalence MAP^c^

Classes 1 2 3 4 5 1 2 3 4 5

Three .82 4486.24 4537.94 .44 .18 .38 - - .92 .94 .91 - -

**Four .80 4484.21 4555.60 .42 .16 .19 .24 - .93 .96 .85 .83 -**

Five .76 4496.07 4597.80 .28 .15 .19 .18 .20 .82 .95 .86 .86 .79

a BIC = Bayesian Information Criterion, b ICL = Integrated Classification Likelihood, c Maximum aposterior probability of class membership

**Bold solution was chosen as providing the most robust and clinically meaningful distribution of classes**

Table S5 *Correlations between Indicator Variables and Regression of Indictor Variables on the Broader LPA Solution – Main Cohort*

Vineland ADOS-2 SCQ CBCL Researcher CBCL R-squared

CSS ADHD ADHD Anxiety

Mullen ELC 0.66 -0.16 -0.49 -0.40 -0.35 -0.42 0.76^***^

Vineland ABC -0.19 -0.56 -0.47 -0.22 -0.38 0.55^***^

ADOS-2 CSS 0.24 0.23 0.23 0.21 0.09^**^

SCQ 0.66 0.12 0.61 0.73^***^

CBCL ADHD 0.28 0.55 0.54^***^

Researcher ADHD 0.20 0.09^*^

CBCL Anxiety 0.57^***^

* p<.05, ** p<.01, *** p<.001

ELC = Mullen Early Learning Composite, ABC = Vineland Adaptive Behavior Composite, ADOS-2 CSS = ADOS-2 Calibrated Severity Score, SCQ = Social Communication Questionnaire, CBCL = Child Behavior Checklist

Table S6 *Summary of the Latent Profile Analysis (LPA) Models using only Autism and ADHD Measures – Main Cohort*

Number of Entropy BIC^a^ ICL^b^ Relative prevalence MAP^c^

Classes 1 2 3 4 1 2 3 4

Two .70 2147.07 2200.48 .64 .36 - - .93 .89 - -

**Three .73 2133.80 2209.61 .29 .17 .54 - .84 .96 .87 -**

Four .70 2155.32 2263.80 .30 .17 .07 .47 .85 .95 .69 .79

a BIC = Bayesian Information Criterion, b ICL = Integrated Classification Likelihood, c Maximum aposterior probability of class membership

**Bold solution was chosen as providing the most robust and clinically meaningful distribution of classes**

Table S7 *3 Year Developmental and Behavioural Characteristics by LPA Outcome Class including only Autism and ADHD Measures – Main Cohort*

**HBC^1^ SBC^2^ TB^3^ ANOVA Post-hocs**

**class** **class class**

*N = 20 N = 72 N = 37*

**Measure** *M (SD) M (SD) M (SD)*

*Measures conceptually similar to those used to derive Miller et al. (2020) outcome classes*

ADOS-2 CSS^b^ 2.95 (2.22) 1.99 (1.37) 1.83 (1.18) F(120,2) = 3.90^*^ 1 > 2, 3

SCQ^d^ 19.99 (6.49) 4.40 (2.32) 1.41 (0.92) F(110,2) = 226.16^***^ 1 > 2, 3; 2 > 3

CBCL ADHD^d^ 67.25 (6.49) 53.20 (4.06) 50.00 (0.00) F(113,2) = 125.50^***^ 1 > 2, 3; 2 > 3

Researcher ADHD^e^ 12.67 (2.37) 11.81 (2.25) 10.77 (2.05) F(105,2) = 3.38^*^

*Other measures used in the broader LPA analysis*

Mullen ELC^a^ 93.71 (16.04) 112.65 (18.41) 123.94 (15.14) F(113,2) = 17.61^***^ 2, 3 > 1; 3 > 2

Vineland ABC ^a^ 83.24 (11.43) 95.38 (10.73) 101.77 (9.73) F(104,2) = 16.67^***^ 2, 3 > 1; 3 > 2

CBCL Anxiety^d^ 66.56 (9.75) 52.83 (5.14) 50.80 (2.30) F(113,2) = 48.71^***^ 1 > 2, 3; 2 > 3

*Other measures (not used in the broader LPA analysis)*

SRS-2 71.88 (12.37) 46.43 (6.24) 42.53 (3.90) F(104,2) = 107.37^***^ 1 > 2, 3

ADI-Social 12.19 (7.04) 2.08 (2.51) 0.83 (1.03) F(118,2) = 89.24^***^ 1 > 2, 3

ADI-Comm 9.11 (4.77) 2.06 (2.93) 0.53 (1.00) F(118,2) = 57.46^***^ 1 > 2, 3; 2 > 3

ADI-RRB 4.37 (1.92) 0.77 (1.17) 0.19 (0.62) F(118,2) = 83.13^***^ 1 > 2, 3

*** p<.05, ** p<.01, ***p<.001; 1, 2, 3 – Class labels for ANOVA Tukey-Kramer corrected post-hocs;

a standard score, b ADOS-2 Calibrated Severity Score, c raw score, d T-score

HBC = High Behavioural Concerns; SBC = Slight Behavioural Concerns; TB = Typical Behaviour

ELC = Mullen Early Learning Composite, ABC = Vineland Adaptive Behavior Composite, ADOS-2 CSS = ADOS-2 Calibrated Severity Score, SCQ = Social Communication Questionnaire, CBCL = Child Behavior Checklist, SRS = Social Responsiveness Scale, ADI = Autism Diagnostic Interview, Comm = Communication, RRB = Repetitive and Restricted Behaviours

Table S8 *Summary of the Broader Latent Profile Analysis (LPA) Models – Independent Cohort*

Number of Entropy BIC^a^ ICL^b^ Relative prevalence MAP^c^

Classes 1 2 3 4 5 1 2 3 4 5

Three .91 4830.34 4858.61 .58 .22 .21 - - .97 .97 .93 - -

**Four .89 4764.34 4807.30 .51 .15 .20 .24 - .96 .91 .90 .93 -**

Five .89 4745.07 4791.59 .51 .03 .17 .18 .11 .95 .99 .92 .91 .90

a BIC = Bayesian Information Criterion, b ICL = Integrated Classification Likelihood, c Maximum aposterior probability of class membership

**Bold solution was chosen as providing the most robust and clinically meaningful distribution of classes**

Table S9 *3 Year Developmental and Behavioural Characteristics by Broader LPA Outcome Class – Independent Cohort*

**LDL+HBC^1^ LDL+TB^2^ TDL+HBC^3^ HDL+TB^4^ ANOVA Post-hocs**

**class class class class**

*N = 21 N = 20 N = 27 N = 71*

**Measure** *M (SD) M (SD) M (SD) M (SD)*

*Measures used to derive LPA outcome classes*

Mullen ELC^a^ 68.90 (12.15) 81.20 (12.56) 112.07 (15.92) 121.51 (12.07) F(132,3) = 115.57^***^ 4 > 1, 2, 3; 3 > 1, 2; 2 > 1

Vineland ABC ^a^ 73.89 (10.09) 93.75 (8.40) 94.15 (7.98) 105.71 (7.05) F(130,3) = 83.51^***^ 4 > 1, 2, 3; 2, 3 > 1

ADOS-2 CSS^b^ 3.29 (3.107) 3.75 (2.34) 2.59 (2.29) 1.60 (1.22) F(132,3) = 8.07^***^ 1, 2 > 4

SCQ^c^ 13.22 (8.19) 2.63 (1.98) 10.85 (7.09) 2.46 (2.10) F(129,3) = 40.64^***^ 1, 3 > 2, 4

CBCL ADHD^d^ 63.00 (9.63) 52.22 (3.93) 60.96 (7.94) 51.03 (2.53) F(124,3) = 35.44^***^ 1, 3 > 2, 4

CBCL Anxiety^d^ 60.26 (10.16) 50.44 (0.98) 61.71 (10.23) 50.63 (1.99) F(124,3) = 28.74^***^ 1, 3 > 2, 4

*Other measures (not used in the LPA analysis)*

SRS^d^ 65.16 (13.56) 42.12 (4.72) 57.00 (12.54) 42.43 (4.37) F(122,3) = 48.34^***^ 1 > 2, 3, 4; 3 > 2, 4

ADI-Social^c^ 8.76 (7.08) 2.05 (2.46) 4.22 (4.08) 1.02 (1.33) F(132,3) = 26.96^***^ 1 > 2, 3, 4; 3 > 4

ADI-Comm^c^ 8.91 (4.84) 2.70 (3.16) 5.44 (5.24) 0.84 (1.32) F(132,3) = 35.53^***^ 1 > 2, 3, 4; 3 > 2, 4

ADI-RRB^c^ 3.05 (2.94) 0.55 (0.69) 2.85 (3.09) 0.13 (0.45) F(132,3) = 22.97^***^ 1, 3 > 2, 4

*** p<.05, ** p<.01, ***p<.001

1, 2, 3, 4 – Class labels for ANOVA Tukey-Kramer corrected post-hocs

a standard score, b ADOS-2 Calibrated Severity Score, c raw score, d T-score

LDL+HBC = Low Developmental Level + High Behavioural Concerns; LDL+TB = Low Developmental Level + Typical Behaviour; TDL+ HBC = Typical Developmental Level + High Behavioural Concerns; HDL+TB = High Developmental Level + Typical Behaviour

ELC = Mullen Early Learning Composite, ABC = Vineland Adaptive Behavior Composite, ADOS-2 CSS = ADOS-2 Calibrated Severity Score, SCQ = Social Communication Questionnaire, CBCL = Child Behavior Checklist, SRS = Social Responsiveness Scale, ADI = Autism Diagnostic Interview, Comm = Communication, RRB = Repetitive and Restricted Behaviours

Table S10 *Number and Percentage of Each LPA Class in Atypical Range on 3 Year Characterisation Measures – Independent Cohort*

**LDL+HBC LDL+TB TDL+HBC HDL+TB**

**class class class class**

**Measure** *N = 21 N = 20 N = 27 N = 71*

Mullen ELC < 85 19 (90%) 12 (60%) 0 (0%) 0 (0%)

Vineland ABC < 85 17 (89%) 4 (20%) 3 (11%) 0 (0%)

ADOS CSS > 4 8 (38%) 12 (60%) 7 (26%) 6 (9%)

SCQ score > 12 9 (50%) 0 (0%) 13 (50%) 0 (0%)

CBCL ADHD T-score > 60 11 (58%) 3 (17%) 16 (67%) 3 (5%)

CBCL Anxiety T-score > 60 8 (42%) 0 (0%) 12 (50%) 1 (2%)

LDL+HBC = Low Developmental Level + High Behavioural Concerns; LDL+TB = Low Developmental Level + Typical Behaviour; TDL+ HBC = Typical Developmental Level + High Behavioural Concerns; HDL+TB = High Developmental Level + Typical Behaviour

N/A = Not applicable.

ELC = Mullen Early Learning Composite, ABC = Vineland Adaptive Behavior Composite, ADOS-2 CSS = ADOS-2 Calibrated Severity Score, SCQ = Social Communication Questionnaire, CBCL = Child Behavior Checklist

**Appendix S2**

*Summary of outcome classes in broader LPA in the independent cohort*

ANOVAs and Tukey-Kramer post-hocs for class differences are in line with class identification (Table S9). The LDL+HBC class had elevated autism, ADHD and anxiety scores, low cognitive ability and adaptive function. The LDL+TB class had low cognitive ability and adaptive function and low parent-reported autism, ADHD and anxiety scores, although they had high ADOS scores perhaps reflecting their overall delayed development. The TDL+HBC class had elevated scores on autism, ADHD and anxiety measures but high average cognitive ability and average adaptive function. The HDL+TB class had low autism, ADHD and anxiety scores, above average cognitive ability and average adaptive function. When considered in terms of the proportion of each class falling above or below clinical thresholds (> 1SD for standardised scores and T-scores; above the autism threshold on the ADOS-2 and SCQ^[[1]](#footnote-1)^) the pattern was similar (Table S10).

Table S11 *LPA Outcome Class by Familial History Group and Sex – Independent Cohort*

**LDL+HBC LDL+TB TDL+HBC HDL+TB**

**class class class class**

*N = 21 N = 20 N = 27 N = 71*

*Familial likelihood Group Total N*

TL *N (%)* 0 (0%) 1 (4%) 2 (8%) 22 (88%) *25*

FH-Autism *N (%)*  16 (20%) 11 (13%) 18 (22%) 37 (45%) *82*

FH-Autism + ADHD *N (%)*  5 (16%) 8 (26%) 7 (23%) 11 (35%) *31*

FH-ADHD *N (%)* 0 (0%) 0 (0%) 0 (0%) 1 (100%) *1*

*139*

*Sex N (%) N (%) N (%) N (%) Total N*

Male 17 (81%)^*^ 16 (80%)^*^ 16 (59%) 27 (38%)^*^  *76*

Female 4 (19%) 4 (20%) 11 (41%) 44 (62%) *63*

*139*

LDL+HBC = Low Developmental Level + High Behavioural Concerns; LDL+TB = Low Developmental Level + Typical Behaviour; TDL+ HBC = Typical Developmental Level + High Behavioural Concerns; HDL+TB = High Developmental Level + Typical Behaviour

* Sex differences: Using chi-square tests followed by post-hoc residuals adjusted for sample size of each group: Girls were over-represented in the HDL+TB class (p < .01) and boys were over-represented in the LDL+HBC class (p < .01) and LDL+TB class (p <.05).

Table S12 *Correlations between Indicator Variables and Regression of Indictor Variables on the Broader LPA Solution – Independent Cohort*

**Vineland ADOS-2 SCQ CBCL CBCL R-squared**

**CSS ADHD Anxiety**

Mullen ELC 0.71 -0.32 -0.27 -0.33 -0.22 0.72^***^

Vineland ABC -0.34 -0.59 -0.50 -0.38 0.66^***^

ADOS-2 CSS 0.27 0.20 0.09 0.16^***^

SCQ 0.64 0.56 0.49^***^

CBCL ADHD 0.56 0.46^***^

CBCL Anxiety 0.41^***^

* p<.05, ** p<.01, *** p<.001

ELC = Mullen Early Learning Composite, ABC = Vineland Adaptive Behavior Composite, ADOS-2 CSS = ADOS-2 Calibrated Severity Score, SCQ = Social Communication Questionnaire, CBCL = Child Behavior Checklist, SRS = Social Responsiveness Scale, ADI = Autism Diagnostic Interview, Comm = Communication, RRB = Repetitive and Restricted Behaviours

Table S13 *3 Year Developmental and Behavioural Characteristics by Broader LPA Outcome Class – Combined Sample*

**LDL+HBC^1^ LDL+TB^2^ TDL+SBC^3^ HDL+TB^4^ ANOVA Post-hocs**

**class class class class**

*N = 45 N = 42 N = 58 N = 125*

**Measure** *M (SD) M (SD) M (SD) M (SD)*

*Measures used to derive LPA outcome classes*

Mullen ELC^a^ 75.95 (13.95) 89.43 (11.94) 115.34 (11.56) 125.71 (10.53) F(249,3) = 243.72^***^ 4 > 1, 2, 3; 3 > 1, 2; 2 > 1

Vineland ABC^a^ 78.17 (10.56) 92.31 (8.16) 94.81 (8.54) 105.15 (7.92) F(239,3) = 101.44^***^ 4 > 1, 2, 3; 2, 3 > 1

ADOS-2 CSS^b^ 3.21 (2.61) 2.62 (2.02) 2.30 (1.82) 1.72 (1.29) F(255,3) = 8.02^***^ 1, 2 > 4

SCQ^c^ 14.74 (7.87) 2.87 (2.04) 9.26 (7.27) 2.39 (1.95) F(242,3) = 80.21^***^ 1 > 2, 3, 4; 3 > 2, 4

CBCL ADHD^d^ 63.52 (8.45) 50.84 (2.43) 57.65 (7.22) 51.32 (3.26) F(240,3) = 64.43^***^ 1 > 2, 3, 4; 3 > 2, 4

CBCL Anxiety^d^ 62.10 (10.65) 51.11 (2.65) 58.94 (8.46) 50.33 (1.12) F(240,3) = 55.24^***^ 1, 3 > 2, 4

*Other measures (not used in the LPA analysis)*

SRS^d^ 65.89 (13.12) 42.57 (4.10) 54.87 (12.38) 42.71 (4.27) F(229,3) = 87.54^***^ 1 > 2, 3, 4; 3 > 2, 4

ADI-Social^c^ 9.50 (7.01) 1.83 (2.35) 3.81 (4.63) 1.02 (1.32) F(254,3) = 54.19^***^ 1 > 2, 3, 4; 3 > 4

ADI-Comm^c^ 8.16 (4.75) 2.76 (3.41) 3.91 (4.73) 0.70 (1.19) F(254,3) = 54.14^***^ 1 > 2, 3, 4; 2, 3 > 4

ADI-RRB^c^ 3.34 (2.74) 0.45 (0.67) 2.00 (2.42) 0.22 (0.62) F(254,3) = 44.19^***^ 1 > 2, 3, 4; 3 > 2, 4

*** p<.05, ** p<.01, ***p<.001

1, 2, 3, 4, 5 – Class labels for ANOVA Tukey-Kramer corrected post-hocs

a standard score, b ADOS-2 Calibrated Severity Score, c raw score, d T-score

LDL+HBC = Low Developmental Level + High Behavioural Concerns; LDL+TB = Low Developmental Level + Typical Behaviour; TDL+ SBC = Typical Developmental Level + Some Behavioural Concerns; HDL+TB = High Developmental Level + Typical Behaviour

ELC = Mullen Early Learning Composite, ABC = Vineland Adaptive Behavior Composite, ADOS-2 CSS = ADOS-2 Calibrated Severity Score, SCQ = Social Communication Questionnaire, CBCL = Child Behavior Checklist, SRS = Social Responsiveness Scale, ADI = Autism Diagnostic Interview, Comm = Communication, RRB = Repetitive and Restricted Behaviours

Table S14 *Number and Percentage of Each LPA Class in Atypical Range on 3 Year Characterisation Measures – Combined Sample*

**LDL+HBC LDL+TB TDL+SBC HDL+TB**

**class class class class**

**Measure** *N = 45 N = 42 N = 58 N = 125*

**____________________________________________________________________________________________________________________**

Mullen ELC < 85 29 (69%) 12 (29%) 0 (0%) 0 (0%)

Vineland ABC < 85 31 (78%) 9 (21%) 3 (6%) 3 (3%)

ADOS CSS > 4 17 (40%) 12 (29%) 14 (25%) 16 (14%)

SCQ score > 12 25 (64%) 0 (0%) 14 (29%) 0 (0%)

CBCL ADHD T-score > 60 28 (70%) 2 (5%) 19 (39%) 8 (7%)

CBCL Anxiety T-score > 60 22 (55%) 1 (3%) 20 (41%) 0 (0%)

LDL+HBC = Low Developmental Level + High Behavioural Concerns; LDL+TB = Low Developmental Level + Typical Behaviour; TDL+ SBC = Typical Developmental Level + Some Behavioural Concerns; HDL+TB = High Developmental Level + Typical Behaviour

ELC = Mullen Early Learning Composite, ABC = Vineland Adaptive Behavior Composite, ADOS-2 CSS = ADOS-2 Calibrated Severity Score, SCQ = Social Communication Questionnaire, CBCL = Child Behavior Checklist

**Appendix S3**

*Summary of outcome classes in broader LPA in combined sample*

Four- and five-class solutions had similar entropy values (0.83 and 0.83, respectively) and the best fit statistics (BIC = 8695.85, ICL = 8826.28 and BIC = 8660.67, ICL = 8809.22, respectively). We chose the four-class solution as providing the most robust and clinically meaningful distribution of classes, with a minimum class size comprising 16.3% (*n* = 45) of the sample and average MAP values for each class all > 0.84. Table S16 shows the correlations between the class indicator variables and R-squared values from regressing each indicator onto the set of classes.

Scores of the four classes on the outcome measures used to derive the classes are shown in Table S13 and Figure S2. Based on the pattern across the measures we labelled the classes: *Low Developmental Level + High Behavioural Concerns* (LDL+HBC; *n* = 45, 17%), *Low Developmental Level + Typical Behaviour* (LDL+TB; *n* = 42, 25%), *Typical Developmental Level + Some Behavioural Concerns* (TDL+SBC; *n* = 58, 34%), and *High Developmental Level + Typical Behaviour* (HDL+TB; *n* = 125, 46%). ANOVAs and Tukey-Kramer post-hocs for class differences are in line with class identification (Table S13). The LDL+HBC class had elevated autism, ADHD and anxiety scores, low cognitive ability and adaptive function. The LDL+TB class had slightly low cognitive ability and adaptive function but not elevated scores on autism, ADHD and anxiety measures. The TDL+SBC class had slightly elevated scores on ADHD and anxiety measures and on the SCQ but not the ADOS or SRS and high average cognitive ability and average adaptive function. The HDL+TB class had low autism, ADHD and anxiety scores, above average cognitive ability and average adaptive function. When considered in terms of the proportion of each class falling above or below clinical thresholds (> 1SD for standardised scores and T-scores; above the autism threshold on the ADOS-2 and SCQ) the pattern was similar (Table S14).

Twenty eight children met diagnostic criteria for autism – 19 were identified in the LDL+HBC class, 8 in the TDL+SBC class and 1 in the LDL + TB class. Table S15 shows the association between the derived classes and the autism and ADHD FH sampling frame and sex. All but one child in the LDL+HBC and LDL+TB classes were from the family history groups. Boys were significantly over-represented in the LDL+HBC and LDL+TB classes and girls in the HDL+TB class.

Table S15 *LPA Outcome Class by Familial History Group and Sex – Combined Sample*

**LDL+HBC LDL+TB TDL+SBC HDL+TB**

**class class class class**

*N = 45 N = 42 N = 58 N = 125*

*Familial likelihood Group Total N*

TL *N (%)*  0 (0%) 3 (6%) 7 (15%) 37 (79%) *47*

FH-Autism *N (%)* 31 (21%) 27 (18%) 33 (22%) 58 (39%) *149*

FH-Autism + ADHD *N (%)*  12 (26%) 9 (19%) 12 (26%) 14 (30%) *47*

FH-ADHD *N (%)* 2 (7%) 3 (11%) 6 (22%) 16 (59%) *27*

*270*

*Sex N (%) N (%) N (%) N (%) Total N*

Male 32 (71%)^*^ 29 (69%)^*^ 31 (53%) 55 (44%) *147*

Female 13 (29%) 13 (31%) 27 (47%) 70 (56%)^*^ *123*

*270*

LDL+HBC = Low Developmental Level + High Behavioural Concerns; LDL+TB = Low Developmental Level + Typical Behaviour; TDL+ SBC = Typical Developmental Level + Some Behavioural Concerns; HDL+TB = High Developmental Level + Typical Behaviour

* Sex differences: Using chi-square tests followed by post-hoc residuals adjusted for sample size of each group: Girls were over-represented in the HDL+TB class (p < .01) and boys were over-represented in the LDL+HBC class (p < .05)

Table S16 *Correlations between Indicator Variables and Regression of Indictor Variables on the Broader LPA Solution – Combined Sample*

**Vineland ADOS-2 SCQ CBCL CBCL R-squared**

**CSS ADHD Anxiety**

Mullen ELC 0.67 -0.28 -0.35 -0.36 -0.28 0.75^***^

Vineland ABC -0.28 -0.57 -0.50 -0.35 0.56^***^

ADOS-2 CSS 0.25 0.22 0.13 0.09^***^

SCQ 0.67 0.57 0.50^***^

CBCL ADHD 0.56 0.45^***^

CBCL Anxiety 0.41^***^

* p<.05, ** p<.01, *** p<.001

ELC = Mullen Early Learning Composite, ABC = Vineland Adaptive Behavior Composite, ADOS-2 CSS = ADOS-2 Calibrated Severity Score, SCQ = Social Communication Questionnaire, CBCL = Child Behavior Checklist

Figure S1 *Profile on Indicator Variables of LPA Outcome Classes – Independent Cohort (Mean + SE bars)*

Figure S2 *Profile on Indicator Variables of LPA Outcome Classes – Combined Sample (Mean + SE bars)*

**References**

Begum-Ali, J., Goodwin, A., Mason, L., Pasco, G., Charman, T., Johnson, M. H., Jones, E. J. H., & STAARS Team. (2022). Altered theta-beta ratio in infancy associates with family history of ADHD and later ADHD-relevant temperamental traits. *Journal of Child Psychology and Psychiatry, 63(9)*, 1057–1067. <https://doi.org/10.1111/jcpp.13563>

Begum-Ali, J., Gossé, L. K., Mason, L., Pasco, G., Charman, T., Johnson, M. H., Jones, E. J. H., & STAARS Team. (2023). Infant sleep predicts trajectories of social attention and later autism traits. *Journal of Child Psychology and Psychiatry*. https://doi.org/10.1111/jcpp.13791

Conners CK. (2008). *Conners 3rd edition*. Toronto: Multi-Health Systems Inc.

Conners CK. (2009). *Conners Early Childhood Manual.* NY: Multi-Health Systems Inc.

Conners CK., Erdhardt, D., Sparrow, E. (1999). *Conners Adults ADHD Ratings Scales*

*(CAARS).* NY: Multi-Health Systems Inc.

Goodman, R., Ford, T., Richards, H., Gatward, R., & Meltzer, H. (2000). The Development and Well-Being Assessment: description and initial validation of an integrated assessment of child and adolescent psychopathology. *Journal of Child Psychology & Psychiatry, 41(5)*, 645-655.

Russell, G., Rodgers. L. R., Ukoumunne, O. C., & Ford, T. (2014). Prevalence of parent-reported ASD and ADHD in the UK: findings from the Millennium Cohort Study. *Journal of Autism & Developmental Disorders, 44(1)*, 31-40. doi: 10.1007/s10803-013-1849-0

Simonoff, E., Pickles, A., Charman, T., Loucas, T., Chandler, S., & Baird, G. (2008). Psychiatric disorders in children with autism spectrum disorders: Prevalence, comorbidity and associated factors in a population-derived sample. *Journal of the American Academy of Child and Adolescent Psychiatry, 47*, 921-929.

1. We used the > 12 threshold on the SCQ as per for 3 year olds (Corsello et al., 2007) [↑](#footnote-ref-1)
