# Supplementary material for: Three year outcomes in infants with a family history of autism and/or attention deficit hyperactivity disorder
Source: JCPP Adv. 2023 Aug 2;3(4):e12189. doi: 10.1002/jcv2.12189 (PMC10694531; doi:10.1002/jcv2.12189)
Supplement: Supplementary file 3 — Figure S2 [file JCV2-3-e12189-s001.pdf]

Figure S2 *Profile on Indicator Variables of LPA Outcome Classes – Combined Sample (Mean + SE bars)*

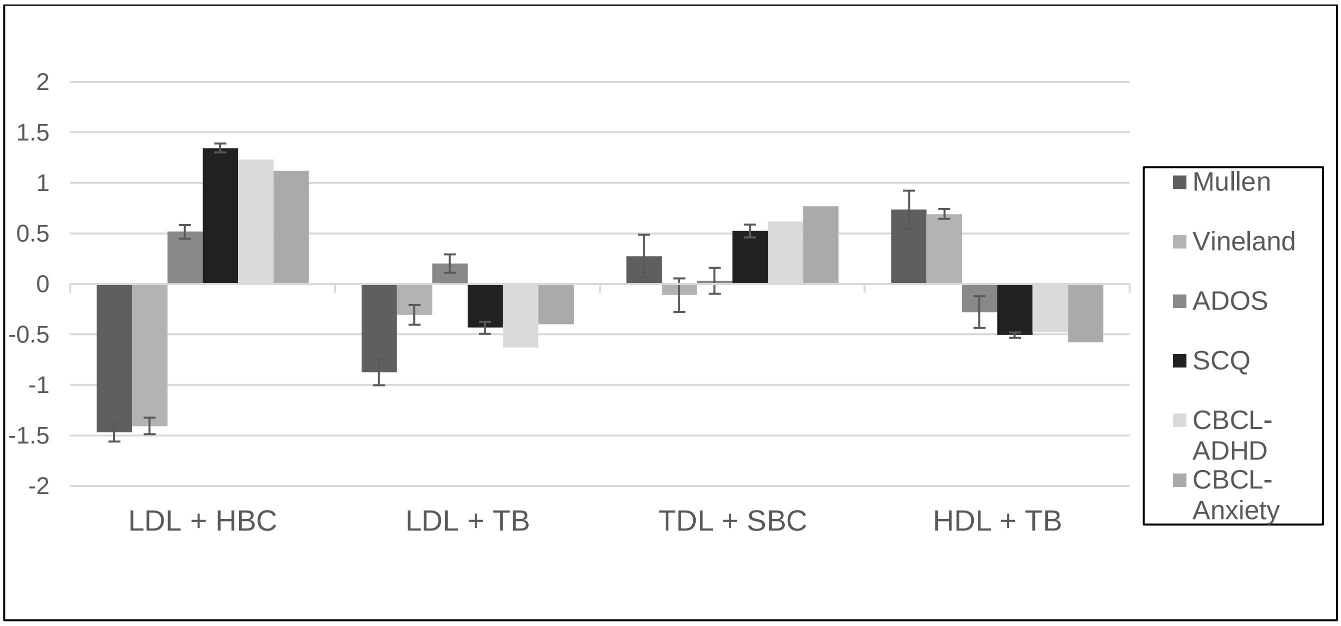

LDL+HBC = Low Developmental Level + High Behavioural Concerns; LDL+TB = Low Developmental Level + Typical Behaviour; TDL+ SBC = Typical Developmental Level + Some Behavioural Concerns; HDL+TB = High Developmental Level + Typical Behaviour

Mullen = Mullen Early Learning Composite, Vineland = Vineland Adaptive Behavior Composite, ADOS = ADOS-2 Calibrated Severity Score, SCQ = Social Communication Questionnaire, CBCL-ADHD = CBCL ADHD subscale, CBCL-Anxiety = CBCL Anxiety subscale

Note: Y-Axis scale is z-score derived separately for each measure from the current sample so all measures are similarly scaled to provide a profile across the measures.
